# The ‘Anthropocene’: *alea iacta est*: The proposal of the ‘Anthropocene’ as a new geological epoch has been submitted for formalization

**DOI:** 10.1038/s44319-024-00065-1

**Published:** 2024-01-22

**Authors:** Valentí Rull

**Affiliations:** 1 Botanic Institute of Barcelona, Spanish National Research Council (CSIC), Pg. del Migdia s/n, 08038 Barcelona, Spain; 2 Institut Català de Paleontologia Miquel Crusafont (ICP-CERCA), Universitat Autònoma de Barcelona, c/ Columnes s/n, 08193 Cerdanyola del Vallès, Barcelona, Spain

**Keywords:** Evolution & Ecology, History & Philosophy of Science

## Abstract

The proposal of the ‘Anthropocene’ as a new geological epoch, characterized by the anthropization of the Earth System, has finally been submitted for formalization.

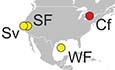

Almost two and a half decades after its first appearance, the term ‘Anthropocene’ is widely used by many scholars as if it was already a well-defined formal epoch of the Geological Time Scale (GTS) but it is still an informal term and its precise definition remains uncertain. Rigor is as important in geology as in any other research discipline, and scientific terms and concepts are therefore subjected to a process of formalization.

Rigor is as important in geology as in any other research discipline, and scientific terms and concepts are therefore subjected to a process of formalization.

The units of the GTS are represented in the International Chronostratigraphic Chart (ICC). Without the ICC, it would not be possible to understand the geological history of our planet or the origin and evolution of life. Such a fundamental framework therefore requires high scientific accuracy. Any new unit to be added has to meet the requirements of the International Stratigraphic Guide (ISG) and must be approved by the International Commission on Stratigraphy (ICS; https://stratigraphy.org/) and ratified by the International Union of Geological Sciences (IUGS; https://www.iugs.org/) (Rull, 2017). This process is similar to the addition of a new element to the Periodic Table of Elements (PTE), which is overseen by the International Union of Pure and Applied Chemistry (IUPAC). The proposition to add the ‘Anthropocene’ as a new geological epoch was evaluated by the Anthropocene Working Group (AWG; http://quaternary.stratigraphy.org/working-groups/anthropocene/), which recently submitted a proposal to the ICS Subcommission of Quaternary Stratigraphy (SQS; http://quaternary.stratigraphy.org/) for approval, as a first step towards formalization.

## In a nutshell

Twenty-three years ago, the Danish environmental chemist Paul Crutzen and the American ecologist Eugene Stoermer coined the term ‘Anthropocene’ to emphasize that the global consequences of human activities have surpassed the range of variability of the Holocene—the epoch since the end of the last glaciation—which would justify the definition of a new geological epoch (Crutzen and Stoermer, 2000). Notably, the term to designate this new unit implicitly suggested the rank of an epoch, as the suffix ‘-cene’ is reserved for the epochs of the Cenozoic era, such as Paleocene, Miocene or Pleistocene.

In 2009, the AWG, led by the British geologists Jan Zalasiewicz (until 2019) and Colin Waters, was formed to analyze Crutzen and Stoermer’s proposal. However, it has been criticized by influential stratigraphers, including the ICS Secretary General, the British geologist Philip Gibbard, and the IUGS Secretary General, the American geologist Stanley Finney, who are directly involved in the approval/ratification of the AWG proposal (Finney, 2014; Gibbard and Walker, 2014; Edwards, 2015; Finney and Edwards, 2015).

One of the main critiques is that the AWG uses environmental criteria to define this new epoch, but a valid chronostratigraphic unit must be defined on the basis of distinct and characteristic rock bodies as the only available evidence for measuring geological time. According to the ISG criteria, the first step is to locate the rock strata that characterize the new unit and the particular features—the stratigraphic markers—that differentiate it from the underlying unit. Then, the base of the new unit is dated using geological methods to provide the chronological framework. This body of evidence is known as the Global Stratotype Section and Point (GSSP), or more popularly the “golden spike”. Without a GSSP, it is not possible to measure geological time and the definition of a new chronostratigraphic unit would therefore make no sense. Like a sand glass with no sand, time passes but it cannot be measured.

Without a GSSP, it is not possible to measure geological time and the definition of a new chronostratigraphic unit would therefore make no sense.

The GSSP for the ‘Anthropocene’ remains undefined though. In 2016, the AWG members voted that the ‘Anthropocene’ starting should be placed in the mid-20^th^ century, coinciding with the Great Acceleration when many indicators of Earth’s anthropization experienced an abrupt increase (Head et al, 2022). The most suitable stratigraphic markers would be radioactive fallout, mainly plutonium (^239^Pu) and radiocarbon (^14^C), which were generated by atomic tests during the 1940s and 1950s (Zalasiewicz et al, 2017). The AWG defined a specific date and a set of stratigraphic markers based on environmental considerations before identifying the GSSP, which is contrary to ISG rules.

This proposal was questioned, not only because of the procedure but also because it dismissed other possible starting points. Indeed, in the original proposal, Crutzen and Stoermer postulated that the ‘Athropocene’ could encompass the last centuries or the last millennia, even the whole Holocene, which began 11,700 years ago. Numerous other studies have proposed a wide range of dates within this timeframe and have emphasized the diachronic (nonsynchronic) nature of human impact across the globe (Lewis and Maslin, 2015). Another critique was that the sedimentary record accumulated during barely 70 years is insufficient for characterizing a geological epoch.

Nonetheless, the AWG decisions were confirmed in 2019 and the task group concentrated on identifying the GSSP that would represent this time period, that is, a rock body that meets the preestablished conditions.

## Latest developments

After a thorough review of the available evidence (Waters et al, 2018), the AWG concluded that the most suitable candidates for the ‘Anthropocene’ GSSP were paleoarchives able to provide high-resolution—annual or seasonal— records from the 20th century, such as annually laminated (varved) sediments from lakes and anoxic marine basins; annual growth rings from trees and corals; or accumulation layers from polar ice caps. The most suitable markers were proposed to be the previously mentioned radionuclides, fly ash, heavy metals, biotic turnovers and anthropogenic introductions, among others (Table 1).

**Table 1:** The localities of Fig. 1, with indications of the type of archive, the date suggested for the beginning of the ‘Anthropocene’ at each site (A-onset), the thickness of the ‘Anthropocene’ sediments (A-thick) in cm, and the stratigraphic markers.

Site	Map	Archive	A-onset	A-thick	Stratigraphic markers
East Gotland, Baltic Sea	EG	Anoxic marine basin	1956 ± 4	26.5	LT, ^239^Pu, ^241^Am
San Francisco, USA	SF	Estuary	Mid-20^th^	230 (?)	Unclear
Searsville, USA	Sv	Lake	1948	366	^239^Pu, SCPs, Pb, BTIs
Crawford, Canada	Cf	Lake	1950	15.6	^239^Pu, SCPs, δ^15^N, BTIs
Sihailongwang, China	Sl	Lake	1953	8.8	LT, ^239^Pu, ^129^I,^14^C, SCPs, PAHs, δ^13^C
Flinders, Australia	Fl	Coral reef	1958	36.9	^239^Pu, ^14^C, Sr/Ca, δ^18^O, δ^15^N
West Flower Garden, USA	WF	Coral reef	1957	28.4	^14^C, ^239^Pu
Palmer, Antarctica	Pm	Ice sheet	1952	3490	^239^Pu, SCPs
Ernesto, Italia	Er	Cave speleothem	1960 ± 3	0.4	^14^C, S
Śnieżka, Poland	Sk	Peatland	1950-1955	39.5-44.5	^239^Pu, ^14^C, BTIs
Beppu, Japan	Bp	Bay	1953	64.6	LT, ^239^Pu, ^210^Pb, δ^15^N
Vienna, Austria	Vn	Urban anthropogenic deposits	1945-1959	30	^239^Pu, AAs, HD

*AAs* anthropogenic artifacts, *BTIs* biotic turnovers/anthropogenic introductions, *HD* historical documentation, *LT* lithology, *SCPs* spheroidal carbonaceous particles (fly ash). Data from Waters et al (2023).

**Figure 1:**
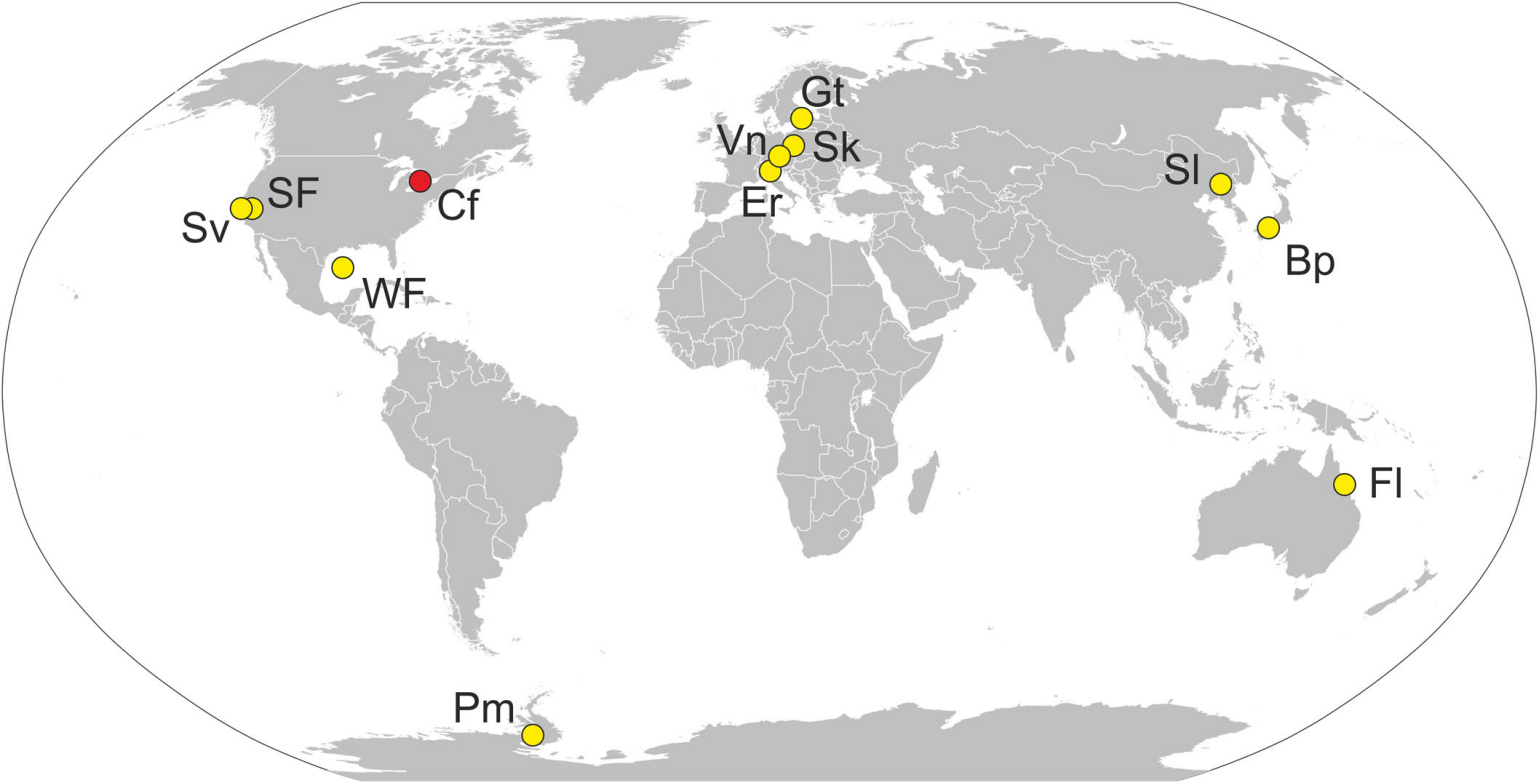
The 12 localities selected by the AWG to determine the most suitable GSSP for the ‘Anthropocene’. The locality selected by the AWG as the best GSSP candidate (Crawford Lake; Cf) is highlighted in red. Redrawn from Waters et al (2023).

Combining the better suited archives and stratigraphic markers, a total of 12 localities around the world were selected for a more intensive study as GSSP candidates (Fig. 1). Using these criteria, the ‘Anthropocene’ was tentatively dated between 1945 and 1968, with most dates occurring in the 1950s (Table 1). After a detailed site-by-site analysis, the AWG announced in July 2023 that the best GSSP candidate was the Canadian Crawford Lake. The bomb test signal (^239^Pu) is clearly visible in the sediments at approximately 15 cm depth, which corresponds to 1950. This boundary is also marked by an enhanced sediment supply from the basin caused by the rapid industrialization of the surrounding area, along with an abrupt decline in elm pollen due to a documented widespread disease of this tree (McCarthy et al, 2023).

The most suitable stratigraphic markers would be radioactive fallout, mainly plutonium (^239^Pu) and radiocarbon (^14^C), which were generated by atomic tests during the 1940s and 1950s.

Some critics, especially the American geologist and former ICS member Lucy Edwards, have argued that barely a few centimeters of unconsolidated lake sediments can easily be mixed or removed—even the whole lake could dry out— which would irreversibly eradicate the ‘Anthropocene’ GSSP (Perkins, 2023). However, the final proposal, still unpublished, proposes the above quoted Crawford Lake GSSP to define the ‘Anthropocene’.

## Further complications

A few years ago, a new possibility emerged that may challenge the progress made by the AWG during the last decade: a group of stratigraphers suggested that the ‘Anthropocene’ could be defined as an event, rather than an epoch (Gibbard et al, 2022). This could affect the formalization process, as this group includes the most influential ICS/IUGS critics quoted above.

… a group of stratigraphers suggested that the ‘Anthropocene’ could be defined as an event, rather than an epoch.

A geological event is a time-transgressive concept that does not need to be homologated using a fixed point in time and is therefore able to accommodate the spatiotemporal heterogeneity characteristic of human impact on Earth. An event is not a minor geological feature and can imply fundamental global transformations. For example, the Great Oxidation Event (GOE) radically changed the course of evolution and enabled the development of multicellular life and the colonization of land. The GOE was not a point in time but rather a gradual process lasting approximately 300 million years (2400–2100 Ma).

According to Gibbard et al (2022), an ‘Anthropocene Event’ could incorporate a far broader range of transformative diachronic anthropogenic practices than an ‘Anthropocene Epoch’. The AWG replied that such an event would include all kinds of human activities that occurred during the past 50 millennia and would obscure the recent abrupt planetary change, which is what an epoch wants to emphasize. In addition, they recall that the suffix ‘-cene’ characterizes Cenozoic epochs and is therefore inappropriate for naming an event (Head et al, 2023).

## The final proposal

The AWG proposal was submitted to the ICS on October 31, 2023 and is now under consideration. Within the ICS, the first instance is the SQS—which is led by two relevant AWG members, Zalasiewicz and the Canadian geologist Martin Head—and the second instance is the ICS Executive, where Gibbard is the Secretary General. In both cases, a minimum of 60% majority is needed for approval. This will not necessarily be a quick step, as the SQS should analyze the proposal in detail. If approved, the proposal will be submitted for ratification to the IUGS where Finney, one of the most active critics of the AWG proposal, is the Secretary General.

According to Waters (pers. comm.), the current AWG Chair, none of these steps are guaranteed to pass and there is no preliminary feedback from the ICS, as its Executive prevented the AWG members from discussing the issue with SQS members. Waters also noted that the SQS is not in favor of publishing the submitted proposal, which will be posted soon in the 2023 AWG Newsletter.

## Expectations

There is a real possibility that the ‘Anthropocene’ proposal will not pass, as a number of relevant ICS/IUGS members have repeatedly questioned AWG decisions. Noteworthy, the AWG always reaffirmed its position without reconsidering the questioned points, which did not help to change the opponents’ perspective. What are the alternatives to an eventual rejection?

When asked about these alternatives (Rull, 2018), the AWG members, including Zalasiewicz and Head, were reluctant to modify the current proposal to downgrade the ‘Anthropocene’ to one more Holocene stage/age, as suggested by Gibbard and others (Gibbard et al, 2022). They emphasize that changes associated with the ‘Anthropocene’ are of greater magnitude than those associated with current subdivisions of the Holocene. Zalasiewicz also stated that there is no plan B and that the AWG will remain attached to the ‘Anthropocene’ concept. Regarding the possibility of the ‘Anthropocene’ to be upgraded to an era—actually the ‘Anthropozoic’ (Rull, 2021)—Edwards stated that, curiously, the AWG never considered such an option. Gibbard and Edwards also commented on the survival of the ‘Anthropocene’ term, regardless of the final outcome, in a cultural sense to emphasize the human influence on global environmental issues, a topic that is beyond the competence of geological organisations.

The general impression is that both proponents and opponents of the current ‘Anthropocene’ proposal remain attached to their own positions and are reluctant to change their mind. The AWG has crossed its Rubicon and is waiting for the result of the SQS deliberations. This subcommission may endorse or reject the proposal but can also request modifications. It is important to note that an eventual rejection does not imply the refusal of the ‘Anthropocene’ as a stratigraphic term and concept but of the current AWG proposal. Therefore, a new and different proposition would still be possible. According to Waters (pers. comm.), some SQS members have published strongly in favor of the AWG proposal and others strongly against, and the result is uncertain, especially if we consider that a 60% majority is required. *Alea iacta est*.

## Societal impact

The ‘Anthropocene’ concept has been adopted by a wide range of disciplines—philosophy, sociology, politics, environmental activism and so on—with different meanings, including an expression of modernity, an attack on Earth’s biosphere, a biological imperative inherent to our own species, a consequence of global capitalism or the decoupling between environmental health and human welfare (Autin, 2016). In some extremist sectors, the rejection of the ‘Anthropocene’ proposal would be viewed as a ‘negationist’ attitude from geological science. However, as the proposal’s opponents clearly emphasized, any decision to formalize such an epoch or not will neither stop nor aggravate the global environmental problems caused by humankind. Other scientists remain indifferent about formalization and have already adopted the ‘Anthropocene’ as a matter of fact, regardless of the final decision, perhaps trusting that formalization is only a matter of time.

The persistence of all these options creates confusion in the general public and among scholars, who are perplexed about the ‘Anthropocene’ and whether or not it is a scientifically valid term and concept. The media greatly contribute to this misperception by announcing the arrival of the ‘Anthropocene’ every time the AWG announced some progress in their deliberations. For example, in 2016, when the task group voted about the onset and the stratigraphic markers chosen to define the new epoch, and in 2023, when Crawford Lake was selected as the GSSP, the media ran headlines such as ‘welcome to the Anthropocene’ or ‘the Anthropocene is here’.

However, as explained above, the approval and ratification of the ‘Anthropocene’ proposal is not guaranteed. Scholars who are concerned with scientific rigor should therefore clearly explain the current and correct status of the ‘Anthropocene’ proposal, not only in scientific publications but also in the media or via other communication venues. Sowing more confusion about the ‘Anthropocene’ by careless or uninformed scholars and writers will neither benefit the geological sciences nor discussions about the human impact on planet Earth.

Sowing more confusion about the ‘Anthropocene’ by careless or uninformed scholars and writers will neither benefit the geological sciences nor discussions about the human impact on planet Earth.

An interesting observation is that, if the current AWG proposal is approved and ratified by the ICS/IUGS, all humans born before 1950 will have originated in a past geological epoch, the Holocene. This means that more than 310 million humans, almost 4% of the total population, could be considered as genuine Holocene living fossils.

… if the current AWG proposal is approved and ratified by the ICS/IUGS, all humans born before 1950 will have originated in a past geological epoch, the Holocene.

### Supplementary information

Peer Review File
