# Supplementary material for: The ‘Anthropocene’: alea iacta est: The proposal of the ‘Anthropocene’ as a new geological epoch has been submitted for formalization
Source: EMBO Rep. 2024 Jan 22;25(3):939–43. doi: 10.1038/s44319-024-00065-1 (PMC10933251; doi:10.1038/s44319-024-00065-1)
Supplement: Supplementary file 1 — Peer Review File [file 44319_2024_65_MOESM1_ESM.pdf]

EMBO reports

The 'Anthropocene': alea iacta est

Valentí Rull
DOI: 10.15252/embr.202358616

Corresponding author(s): Valentí Rull (vrull@csic.es)

Review Timeline:

Submission Date:	8th Dec 23
Editorial Decision:	12th Dec 23
Revision Received:	13th Dec 23
Accepted:	11th Jan 24

Editor: Holger Breithaupt

Transaction Report: This article was editorially reviewed.
